# Molecular profiling and comprehensive genome-wide analysis of somatic copy number alterations in gastric intramucosal neoplasias based on microsatellite status

**DOI:** 10.1007/s10120-018-0810-5

**Published:** 2018-02-21

**Authors:** Tamotsu Sugai, Makoto Eizuka, Noriyuki Arakawa, Mitsumasa Osakabe, Wataru Habano, Yasuko Fujita, Eiichiro Yamamoto, Hiroo Yamano, Masaki Endoh, Takayuki Matsumoto, Hiromu Suzuki

**Affiliations:** 1 Department of Molecular Diagnostic Pathology, School of Medicine, Iwate Medical University, 19-1, Uchimaru, Morioka, 020-8505 Japan; 2 Department of Pharmacodynamics and Molecular Genetics, School of Pharmacy, Iwate Medical University, 19-1, Uchimaru, Morioka, 020-8505 Japan; 3 Department of Molecular Biology, Sapporo Medical University, School of Medicine, Cyuuouku, Sapporo, 060-0061 Japan; 4 Department of Gastroenterology, Sapporo Medical University, School of Medicine, Cyuuouku, Sapporo, 060-0061 Japan; 5 Division of Gastroenterology, Department of Internal Medicine, School of Medicine, Iwate Medical University, 19-1, Uchimaru, Morioka, 020-8505 Japan

**Keywords:** Comprehensive genomic analysis, Copy number alteration, Gastric intramucosal neoplasia, Microsatellite instability, Mutation

## Abstract

**Background:**

We attempted to identify the molecular profiles of gastric intramucosal neoplasia (IMN; low-grade dysplasia, LGD; high-grade dysplasia, HGD; intramucosal cancer, IMC) by assessing somatic copy number alterations (SCNAs) stratified by microsatellite status (microsatellite stable, MSS; microsatellite instable, MSI). Thus, microsatellite status was determined in 84 tumors with MSS status and 16 tumors with MSI status.

**Methods:**

One hundred differentiated type IMNs were examined using SCNAs. In addition, genetic mutations (*KRAS*, *BRAF*, *PIK3CA*, and *TP53*) and DNA methylation status (low, intermediate and high) were also analyzed. Finally, we attempted to identify molecular profiles using a hierarchical clustering analysis.

**Results:**

Three patterns could be categorized according to SCNAs in IMNs with the MSS phenotype: subgroups 1 and 2 showing a high frequency of SCNAs, and subgroup 3 displaying a low frequency of SCNAs (subgroup 1 > 2 > 3 for SCNA). Subgroup 1 could be distinguished from subgroup 2 by the numbers of total SCNAs (gains and losses) and SCN gains (subgroup 1 > 2). The SCNA pattern of LGD was different from that of HGD and IMC. Moreover, IMNs with the MSI phenotype could be categorized into two subtypes: high frequency of SCNAs and low frequency of SCNAs. Genetic mutations and DNA methylation status did not differ among subgroups in IMNs.

**Conclusion:**

Molecular profiles stratified by SCNAs based on microsatellite status may be useful for elucidation of the mechanisms of early gastric carcinogenesis.

**Electronic supplementary material:**

The online version of this article (10.1007/s10120-018-0810-5) contains supplementary material, which is available to authorized users.

## Introduction

Gastric cancer (GC) remains one of the leading causes of cancer-related death worldwide, despite recent decreases in the incidence and mortality rates associated with this disease [1]. Histological classification of GC is important to understand the characteristics of gastric carcinogenesis [2–5]. Histogenesis of GC has been classified into intestinal and diffuse types, which are thought to involve different molecular pathways [1–6]. This classification is closely associated with not only clinicopathological findings but also molecular alterations in GC. According to this theory, separate molecular pathways should be established in intestinal and diffuse types [1, 6]. However, guidelines for endoscopic therapy recommend that the main target of endoscopic therapy is limited to the differentiated type, confined to intramucosal cancer or shallow depth of submucosal cancer (under 0.5 mm) [1, 6]. Identification of gastric carcinogenesis of differentiated type is important for not only pathological but also clinical aspects. In addition, gastric differentiated type intramucosal neoplasia (IMN) is a heterogeneous entity including low-grade dysplasia (LGA), high-grade dysplasia (HGA), and intramucosal adenocarcinoma (IMA) in terms of histological grading [7]. A classification system for intramucosal neoplasia (LGD, HGD, and IMC) has been developed by the World Health Organization (WHO), with guidelines published in 2011, and has been widely used around the world [6]. However, the association of these neoplastic conditions with molecular alterations has not been fully evaluated.

Recent study has shown that gastric molecular carcinogenesis can be classified into four subgroups, including chromosomal instability (CIN), microsatellite instability (MSI), EBV, and genomically stable (GS) subtypes [8]. The CIN subtype is characterized by intestinal histology, *TP53* mutations, and accumulation of copy number alterations, whereas MSI subtype is closely associated with the microsatellite unstable, CpG island methylation phenotype (CIMP) and *MLH1* silencing [8]. In addition, the EBV subtype may be linked to *PIK3CA* mutations, CIMP (but not MSI status), and *CDKN2A* silencing. Finally, diffuse histology, mutations in *CDH1* and *RHOA*, and *CLDN18*-*ARHGAP* fusions are characteristic factors in the GS subtype [8]. Moreover, this theory may be applicable to advanced cancer [8]. For example, the CIN subtype may not be an appropriate type in intramucosal cancer of the intestinal type, given that the chromosomal accumulation characterizing the CIN type at the molecular level may still not occur in intramucosal cancer. According to this concept, there are additional subgroups, including CIN and non-CIN subtypes, in differentiated IMN.

Alternative molecular classifications have been used worldwide in gastrointestinal carcinogenesis, including microsatellite stable (MSS) and microsatellite instability (MSI) [8–11]. MSS and MSI subtypes are mutually exclusive. However, the MSS subtype is not thought to be a homogeneous entity and may be composed of different molecular alterations [8–10]. This concept enables us to elucidate molecular differences based on MSS and MSI status in IMNs.

In the present study, we aimed to identify the molecular differences in gastric IMNs between MSS and MSI based on stratification of SCNAs. In addition, we examined the associations of other molecular factors (mutations in *TP53*, *BRAF*, *PIK3CA* and *KRAS* and DNA methylation) with SCNAs in gastric IMNs based on MSI and MSS.

## Materials and methods

### Patients

One hundred patients with intramucosal neoplasia (IMN) obtained from gastric endoscopic submucosal dissection (ESD) were enrolled in this study. Clinicopathological findings were recorded according to the general rules for management of the Japanese gastric cancer association [12]. However, IMN was evaluated according to the modified WHO 2010 criteria [6]. Briefly, LGD was characterized by a uniform monolayer of columnar cells with basal nuclei showing minimal atypia. In HGD, nuclear atypia was more frequent, with nuclear pleomorphism, nuclear enlargement, and pseudostratification without stromal invasion. In IMC (differentiated type or intestinal type), there was marked cytological atypia and complex architecture with cribriform groups, irregular branching, glandular anastomosis, and budding of neoplastic cells into the lumen, which were considered representative of stromal invasion [6]. This study was approved by the local ethics committee of Iwate Medical University (Approval Number HGH28-25), and all patients provided informed consent.

### Sampling of the lesions examined in this study

Tumor tissue was obtained from the resected stomach using biopsy forceps within 30 min of resection. Normal gastric mucosa distant from the tumor was removed from the submucosa using scissors, and, as a control, gastric biopsies from patients with IMN with chronic gastritis were included. Tumor tissues for clinicopathological analysis were obtained from a region of the resected stomach adjacent to the site used for molecular analysis (one sample was obtained as a representative sample). In this section, the proportion of tumor cells accounted for at least 50% of the tissue.

### DNA extraction

We stored the fresh tumor and normal samples at − 80 °C until the molecular analysis. DNA was extracted from isolated normal and tumor tissue by sodium dodecyl sulfate (SDS) lysis and proteinase K digestion, followed by a phenol–chloroform procedure.

### MSI analysis

The extracted DNA was amplified by polymerase chain reaction (PCR) with fluorescent dye-labeled primers targeting five microsatellite loci: BAT25, BAT26, D5S346, D2S123, and D17S250. DNA was detected using a DNA sequencer (PRISM 377; Perkin-Elmer Corp., Foster City, CA, USA), and fragment analyses were performed with GeneScan software (Perkin-Elmer Corp.), previously described [13]. According to the NCI criteria [13], MSI-H was defined as instability in at least 2 of the 5 microsatellite loci; MSI-L as instability in only 1 locus; and MSS when none of the loci were shifted. In the present study, tumors with MSI-low and MSS were regarded as MSS.

### Analysis of TP53 and PIK3CA mutations

Mutations in *TP53* (exons 5–8) and *PIC3CA* (exon 9 and 20) were assessed by PCR single-strand conformation polymorphism (PCR-SSCP) analysis and sequencing as previously described [14]. After PCR-SSCP analysis, direct sequencing of the abnormal bands was performed. The PCR products were sequenced by the dideoxy chain termination method.

### Analysis of KRAS and BRAF mutations

Mutations in *KRAS* (exon 2) and *BRAF* (exon 15; V600E) genes were examined using a pyrosequencer (Pyromark Q24; Qiagen NV), as previously described [15]. The primers design used in the present study was previously described [16]. The cutoff value for the mutation assay was 15% mutant alleles.

### Pyrosequencing for evaluation of methylation

We used a second panel method to determine the methylation status as previously described [17, 18]. The DNA methylation status of each gene promoter region was established by PCR analysis of bisulfite-modified genomic DNA (EpiTect Bisulfite Kit; Qiagen) using pyrosequencing for quantitative methylation analysis (Pyromark Q24; Qiagen NV). Briefly, 6 markers (*RUNX3*, *MINT31*, *LOX*, *NEUROG1*, *ELMO1*, and *THBD*) were selected for determination of the genome-wide methylation status [17]. After methylation analysis of a panel of 3 markers (*RUNX3*, *MINT31*, and *LOX*), tumors with hypermethylated epigenomes (HMEs) were defined as those with at least 2 methylated markers. The remaining tumors were examined using 3 markers (*NEUROG1*, *ELMO1*, and *THBD*). Tumors with moderately methylated epigenomes (IMEs) were defined as those with at least 2 methylated markers, and tumors not classified as having HMEs or IMEs were designated as having hypomethylated epigenomes (LMEs).

The cutoff value for the methylation assay was 30% of the tumor cells, as previously reported [15].

### Somatic copy number alteration (SCNA) analysis

Extracted DNA was adjusted to a concentration of 50 ng/μL. All 100 paired samples were assayed using the Infinium HumanCytoSNP-12v2.1 BeadChip (Illumina, San Diego, CA, USA), which contained 299,140 single nucleotide polymorphism (SNP) loci, according to the Illumina Infinium HD assay protocol [19]. BeadChips were scanned using iScan (Illumina) and analyzed using GenomeStudio software (v.2011.1; Illumina). Log R ratio (LRR) and B allele frequency (BAF) data from each sample were exported from normalized Illumina data using GenomeStudio. Data analysis was conducted with KaryoStudio 1.4.3 (copy number variation [CNV] Plugin v3.0.7.0; Illumina). The program was used with default parameters. CNAs were classified by SCNA partition algorithms. LRR 0 indicated a normal diploid region. LRR greater than 0 indicated copy number gains. LRR less than 0 indicated copy number loss of heterozygisity (LOH). BAF values ranged from 0 to 1; homozygous SNPs had BAFs near 0 (A-allele) or 1 (B-allele), whereas heterozygous diploid region SNPs had BAFs near 0.5 (AB genotype). Additionally, LRR and BAF data were used to identify regions of hemizygosity and copy-neutral LOH.

### Statistical analysis

Hierarchical analysis was performed for clustering the samples according to the SCNA pattern in order to achieve maximal homogeneity for each group and the highest difference between groups. The clustering algorithm was set to centroid linkage clustering, the standard hierarchical clustering method used in biological analyses [20]. Briefly, the basic theory is to assemble a set of items (e.g., CNA) into a tree, where items are joined by very short branches if they are very similar to each other and by increasingly longer branches as their similarity decreases. The first step in hierarchical clustering is to calculate the distance matrix between the CNA data. Once this matrix of distances is computed, the clustering begins. Hierarchical processing consists of repeated cycles where the two closest remaining items (those with the smallest distance) are joined by a node/branch of a tree, with the length of the branch set to the distance between the joined items. The two joined items are removed from the list of items being processed and replaced by an item that represents the new branch. The distances between this new item and all other remaining items are computed, and the process is repeated until only one item remains. The process allows us to perform appropriate cluster analysis on our CNA database.

Data obtained for clinicopathological findings, histological features, mutation status, and methylation status based on each subgroup were analyzed using Chi square tests with the aid of Stat Mate-III software (Atom, Tokyo, Japan). If statistical differences between the 3 groups were found, statistical analysis between two groups was further performed using Chi square tests (Stat Mate-III software). Differences in age distributions between the 3 groups were evaluated using the Kruskal–Wallis *H* test with the aid of Stat Mate-III software (Atom). Differences with *p* values of less than 0.05 were considered significant.

## Results

### Molecular classification

IMNs with MSS and MSI phenotypes were identified in 84 and 16 tumors, respectively. Clinicopathological findings based on MSS and MSI status are shown in Table 1.

**Table 1:** Clinicopathological findings of intramucosal neoplasias according to microsatellite status

	Total	MSS	MSI
Total	100	84	16
Age	45–88 (75)	45–88 (73)	57–88 (77)
Men/women	73/27	66/18	7/9
Size (mm)	3–65 (19)	3–64 (16)	10–65 (23)
Locus			
U	18	17	1
M	26	20	6
L	56	47	9
Macroscopic type			
Elevated	55	42	13
Flat	3	3	0
Depressed	42	39	3
Differentiation			
LGD	37	34	3
HGD	36	28	8
IMA	27	22	5
Depth			
Mucosa	100	84	16
Lymphatic invasion (−)	95	82	15
Lymphatic invasion (+)	3	2	1
Vascular invasion (−)		84	16
Vascular invasion (+)		0	0

The vertical line shows copy number alterations, and the horizontal lines denote “relatedness” between samples and SCNAs at the chromosomal loci. We carried out hierarchical clustering analysis based on the SCNA pattern, including gains, LOHs, and copy-neutral LOHs, to examine differences in genetic alterations in samples from patients with IMNs with the MSS and MSI phenotypes.

### Hierarchical clustering analysis based on the copy number alteration patterns in IMNs with the MSS phenotype

**Fig. 1:**
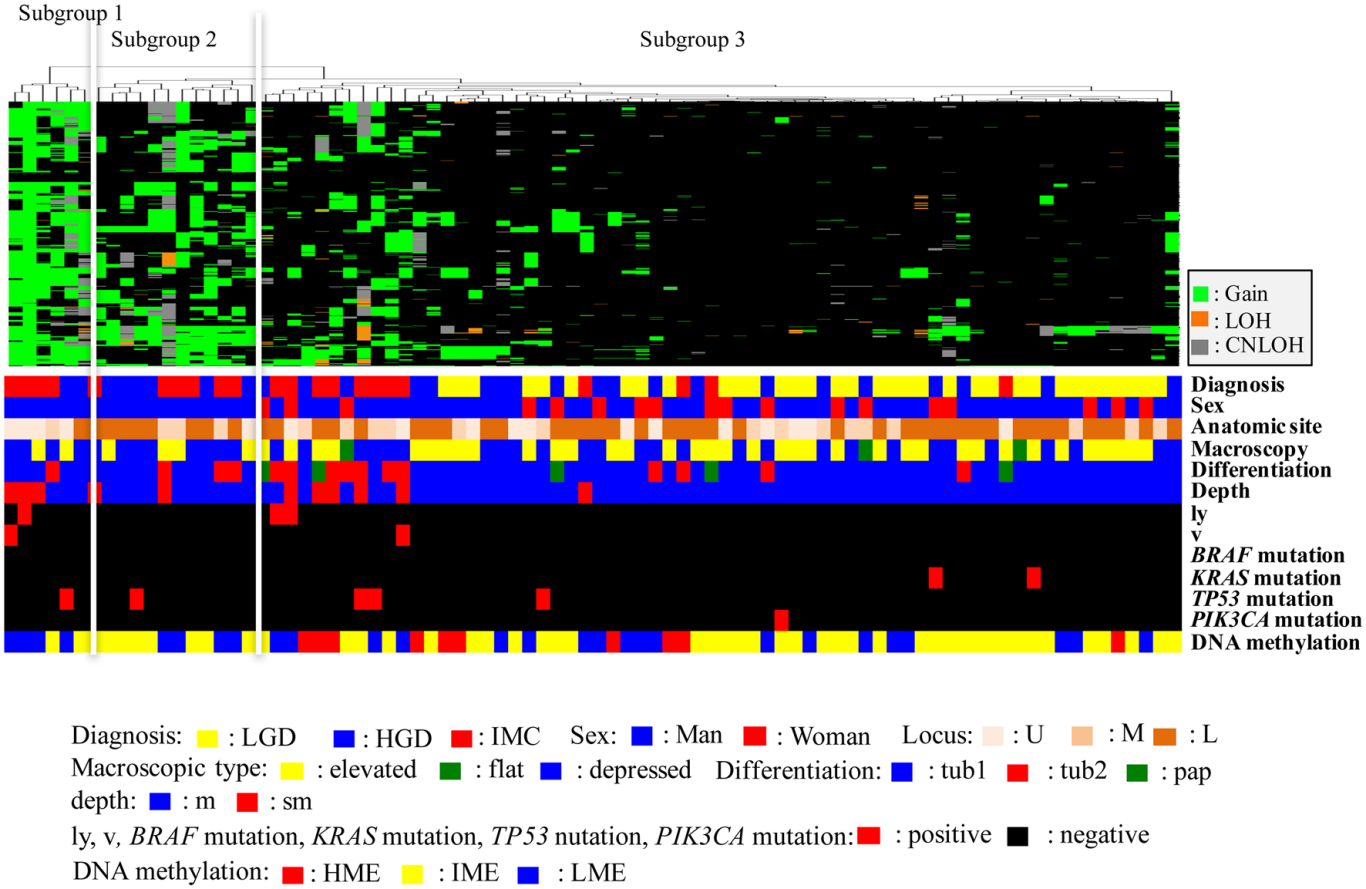
Hierarchical cluster analysis based on somatic copy number alterations in 84 gastric intramucosal neoplasias with the MSS phenotype. *LGD* low-grade dysplasia, *HGD* high-grade dysplasia, *IMC* intramucosal cancer, *U* upper, *m* middle, *L* lower, *tub1* well-differentiated adenocarcinoma, *tub2* moderately differentiated adenocarcinoma, *pap* papillary adenocarcinoma, *ly* lymphatic invasion, *v* venous invasion, *HME* high methylation epigenotype, *IME* intermediate methylation epigenotype, *LME* low methylation epigenotype, *MSI* microsatellite instability, *MSS* microsatellite stable

We could sub-classify IMNs with the MSS phenotype into 3 subgroups according to SCNA patterns (Fig. 1; subgroup 1, 6 tumors; subgroup 2, 12 tumors; subgroup 3, 66 tumors). This classification could be made based on the genetic similarity that characterizes the patterns of SCNAs, including gains, LOH, and copy-neutral LOHs; in particular, the gain patterns between subgroups 1 and 2 were different [20]. Clinical findings in each subgroup categorized based on SCNAs were examined. Although the frequencies of LGD were higher in tumors in subgroup 3 (55.5%; 34/66 tumors) than in subgroups 1 (0/6 tumors) and 2 (0/12 tumors; *p* < 0.01), those of IMC were significantly higher in tumors in subgroups 1 (66.7%; 4/6 tumors) and 2 (50%; 6/12 tumors) than in those in subgroup 3 (18.2%; 12/66 tumors; *p* < 0.01). HGD was commonly found in tumors in subgroups 1 (33.3%; 2/6 tumors), 2 (50%; 6/12 tumors), and 3 (30.3%; 12/66 tumors).

The frequencies of gene mutations (*TP53*, *KRAS*, *BRAF*, and *PIK3CA*) were not different between tumors in subgroups 1 (*TP53*, 1/6, 16.7%; *KRAS*, 0/6; *BRAF*, 0/6; *PIK3CA*, 0/6), 2 (*TP53*, 1/12, 8.3%; *KRAS*, 0/12; *BRAF*, 0/12; *PIK3CA*, 0/12), and 3 (*TP53*, 3/66, 4.5%; *KRAS*, 2/66, 3.0%; *BRAF*, 0/66; *PIK3CA*, 1/66, 1.5%). In DNA methylation, IME was commonly found in all three subgroups (subgroup 1, HME, 0/6; IME, 2/6, 33.3%; LME, 4/6, 66.7%; subgroup 2, HME, 0/12; IME, 8/12, 66.7%; LME, 4/12, 33.3%; subgroup 3, HME, 10/66, 15.2%; IME, 37/66, 56.1%; LME, 19/66, 28.8%). In addition, no *MLH1* methylation was observed in subgroups 1, 2, and 3.

### Copy number alterations between tumors in subgroups 1, 2, and 3 categorized based on SCNAs in IMNs with an MSS phenotype

**Fig. 2:**
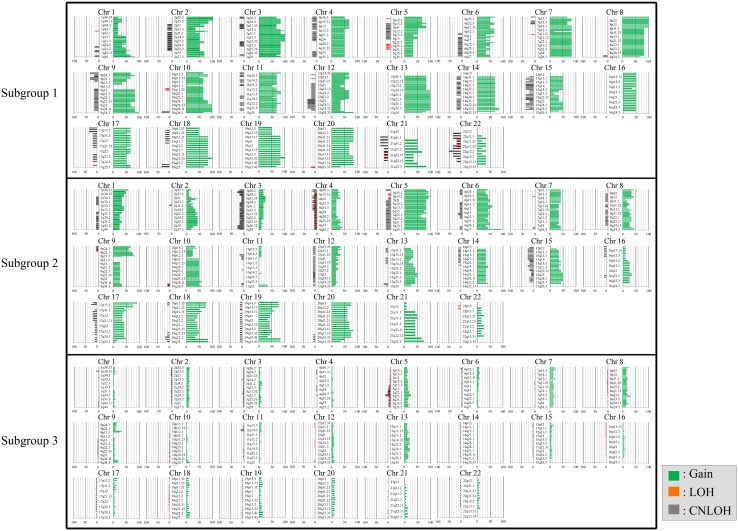
Ideogram of copy number alterations in three subgroups categorized based on somatic copy number alteration patterns in gastric intramucosal neoplasias. Chromosomes are ordered from 1 to 22. The colored horizontal lines represent the frequencies of gains, LOHs, and CNLOHs. Lines on the left indicate losses (red, copy-neutral LOH; gray, LOH), and those on the right (green) indicate gains. **a** Ideogram of copy number alterations in subgroup 1. **b** Ideogram of copy number alterations in subgroup 2. **c** Ideogram of copy number alterations in subgroup 3

The ideograms of subgroups 1, 2, and 3 are depicted in Fig. 2. The mean total number of chromosomal aberrations per patient was 549, with an average of 481 gains (range 338–771), 4 LOHs (range 0–20), and 64 copy-neutral LOHs (range 0–174) in subgroup 1. In addition, the mean total number of chromosomal aberrations per patient was 322, with an average of 269 gains (range 129–402), 5 LOHs (range 0–45), and 48 copy-neutral LOHs (range 0–318) in subgroup 2. In contrast, the mean total number of chromosomal aberrations per patient was 70, with an average of 56 gains (range 0–282), 3 LOHs (range 0–75), and 11 copy-neutral LOHs (range 0–174) in subgroup 3. There were significant differences in the total numbers of SCNAs among subgroups 1, 2, and 3 (*p* < 0.01; *p* < 0.001). Moreover, significant differences in the average number of copy number gains among subgroups 1, 2, and 3 were found (*p* < 0.01; *p* < 0.01). LOH and copy-neutral LOH were common in the three subgroups.

In subgroup 1, regions of gain detected in more than 30% of cases were located at 1p36.11-p36.23, 1q21.1-q44, 1p11.2-p13.2, 2p, 2q, 3p, 3q, 4p, 4q, 5p, 5q, 6p, 6q, 8p, 8q, 13q, 10p, 10q, 7p11.2-22.3, 7q, 9p21.11-q34.3, 9p12-p24.3, 9p12-p24.3, 11p, 11q, 12p, 12q, 15q11-21.3, 15q22.31-q26.3, 16p, 16q, 14q, 17p, 17q, 18p, 18q, 19p, 20p, and 20q; in contrast, in subgroup 2, regions of gain detected in more than 30% of cases were located at 1p, 1q21.1-q21.3, 1q31.1-q32.1, 2p16.1-p16.3, 2q14.1-q35.2, 5q, 5p, 6p, 6q, 7p11.2-p22.3, 7q11-q21.11, 7q21.13-q33, 7q35-q36.3, 8p, 8q21.3, 8q22.3-q24.11, 9p, 10q, 12q13.3, 13q12, 13q14.3-q34, 14q, 15q11.2-q26.3, 17p, 17q, 18q, 18p, 19p12-13.3, 20p, 20q, and 21q11.2-q22.13 (in decreasing order of frequency). No regions of gain in more than 30% of cases were found in subgroup 3. Although regions of copy-neutral LOH detected in more than 30% of cases were found at 9p23-p24.3, 9p21.1-p21.3, 12q23.1-q24.3, 15q12-q13.1, 15q13.3-q24.1, 15q25.1-q26.3, 21q13.1, 17p11.2-p13.3, 21q11.2-q21.3, and 22q11.1-13.31 in subgroup 1, none were observed in subgroups 2 and 3. In addition, no LOHs detected in more than 30% of cases were found. These results are summarized in Supplementary Table 1.

### Differences of copy number alterations between tumors in subgroups 1, 2, and 3 categorized based on SCNAs in IMNs with the MSS phenotype

Next, we examined differences in SCNAs between the three subgroups, as shown in Table 2. Regions of gain detected in more than 50% of cases were selected for comparison of each group. Significant differences in the frequencies of CN gains between subgroups 1 and 2 were detected at 2p24.1-p25.1, 3p13, 3q11.1-q13.32, 3q24-q27.1, 3q28-q29, 8q11.1, 11p15.4, 11q12.2-q14.1, 11q14.3-q25, 11q13.2-q13.4, and 11q22.3. No significant differences in losses (LOH and copy-neutral LOH) were observed between subgroups 1 and 2.

**Table 2:** Significant differences in the frequencies of SCNAs between subgroups 1 and 2 in IMN with the MSS phenotype

	Subgroup 1 *n* = 6 (%)	Subgroup 2 *n* = 12 (%)	*p* value
Gain			
2p24.1-p25.1	6 (100)	1 (8.3)	< 0.01
11q13.2-q13.4, 11q22.3	5 (83.3)	0	< 0.01
3p13, 3q11.1-q13.32, 3q24-q27.1, 3q28-q29	5–6 (83.3–100)	1–2 (8.3–16.7)	< 0.05
8q11.1	5 (83.3)	1 (8.3)	< 0.05
11p15.4, 11q12.2-q14.1, 11q14.3-q25	4–5 (66.7–83.3)	0–1 (0–8.3)	< 0.05
Copy-neutral LOH			
None			
LOH			
None			

There were significant differences in the frequencies of gains at 1p, 1q, 2p, 2q, 3p, 3q, 4p, 4q, 5p, 6p, 6q, 7p, 7q, 8p, 8q, 9p, 9q, 10p, 10q, 11p, 11q, 12p, 12q, 13q, 14q, 16p, 17q, 18q, 19p, 20 ps and 20q between subgroups 1 and 3 (subgroup 1 < 3). In addition, significant differences in the frequencies of copy-neutral LOH at 9p21.1-p21.3, 9p23-p24.3, 12q23.1-q24.31, 15q13.3-q24.1, 15q25.1-q26.3, 17p12-p13.3, 21q11.2-q21.3, 22q13.1, 22q11.21-q11.22, and 22q12.2-q13.31 were observed between tumors in subgroups 1 and 3. Finally, we examined significant differences in the frequencies of SCNAs between subgroups 3 and 1 or 2. Significant differences in copy number gains were observed between subgroups 2 and 3 at 1p, 2q, 4q, 4p, 5p, 5q, 6p, 6q, 9p, 10p, 10q, 12p, 14q, 14p, 15q, 15q, 17p, 17q, 18p, 18q, 19p, 19q, 20p, 20q, and 21q (subgroup 3 < 2). Moreover, there were significant differences in the frequencies of copy-neutral LOH at 3p25.1-p26.1, 3q26.32-q28, 15q11.2, 15q13.2-q15.3, 15q22.2-q22.31, and 17p11.2 between tumors in subgroups 2 and 3. These results are summarized in Supplementary Tables 2 and 3.

### Hierarchical clustering analysis based on the SCNA patterns in IMNs with the MSI-high phenotype

**Fig. 3:**
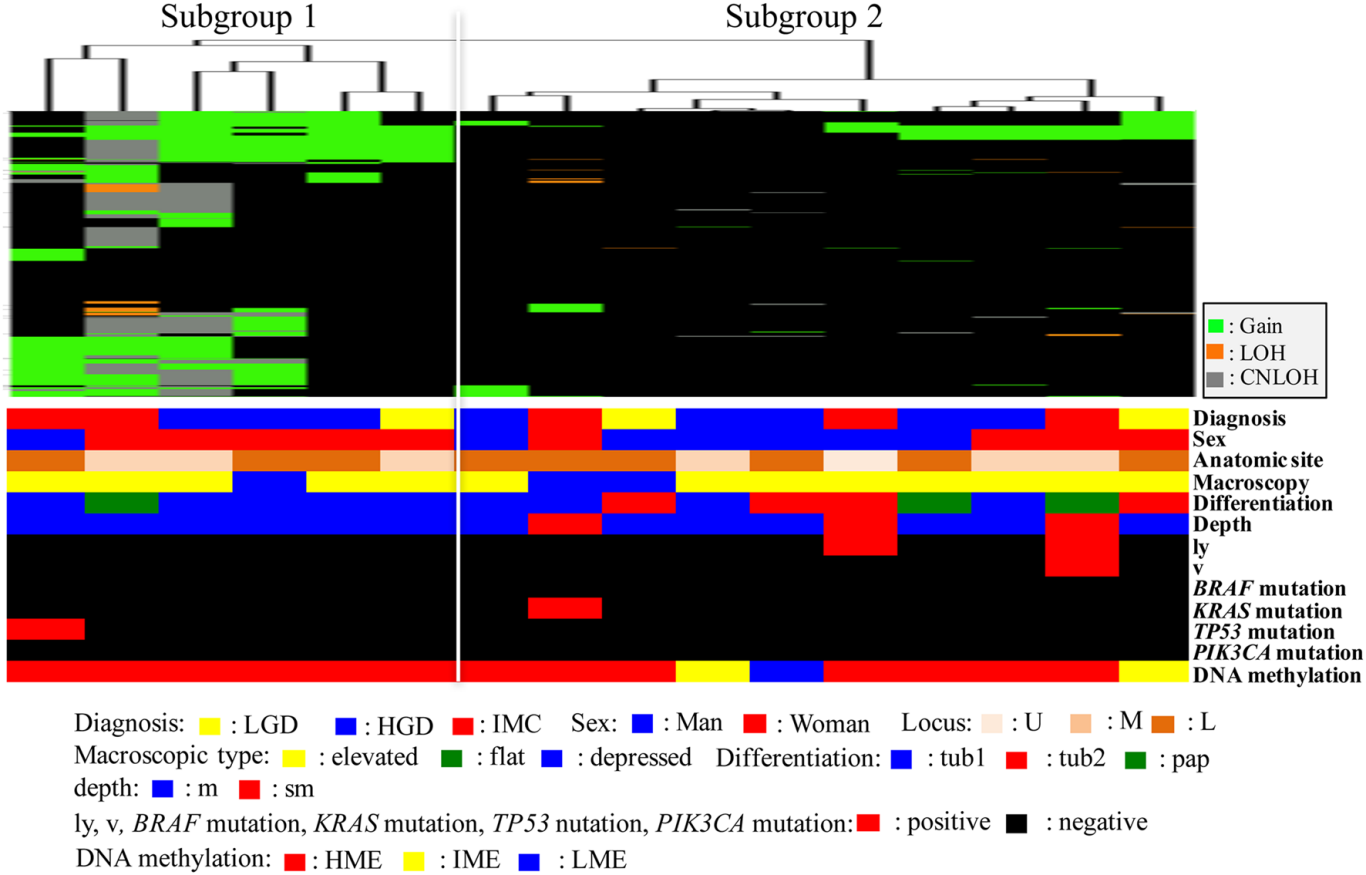
Hierarchical cluster analysis based on somatic copy number alterations in 16 gastric intramucosal neoplasias with the MSI-high phenotype. *LGD* low-grade dysplasia, *HGD* high-grade dysplasia, *IMC* intramucosal cancer, *U* upper, *m* middle, *L* lower, *tub1* well-differentiated adenocarcinoma, *tub2* moderately differentiated adenocarcinoma, *pap* papillary adenocarcinoma, *ly* lymphatic invasion, *v* venous invasion, *HME* high methylation epigenotype, *IME* intermediate methylation epigenotype, *LME* low methylation epigenotype, *MSI* microsatellite instability, *MSS* microsatellite stable

We also performed hierarchical clustering analysis of IMNs with the MSI phenotype based on the SCNA pattern. We sub-classified IMNs with the MSI phenotype into subgroups 1 (6 tumors) and 2 (10 tumors) based on SCNA patterns (Fig. 3).

Clinical findings in each subgroup categorized based on SCNAs were examined. There were no significant differences in the frequencies of clinicopathological findings between subgroups 1 and 2. Significant differences in the frequencies of histological grades were not found in tumors in subgroups 1 (LGD, 1/6; HGD, 3/6; IMC, 2/6) and 2 (LGD, 2/10; HGD, 5/10; IMC, 3/10).

The frequencies of gene mutations (*TP53*, *KRAS*, *BRAF*, and *PIK3CA*) were not different between tumors in subgroups 1 (*TP53*, 1/6, 16.7%; *KRAS*, 0/6; *BRAF*, 0/6; *PIK3CA*, 0/6) and 2 (*TP53*, 0/10; *KRAS*, 0/10; *BRAF*, 1/10, 10%; *PIK3CA*, 0/10). In DNA methylation analysis, HME was commonly found in the three subgroups (subgroup 1, HME, 6/6, 100%; IME, 0/6 and LME, 0/6; subgroup 2, 7/10, 70%; 2/10, 20%; 1/10, 10%). In addition, *MLH1* methylation was found in 12 of 16 IMNs with an MSI-high phenotype (75%). However, there were no differences in methylation rates between subgroups 1 and 2 of IMNs with an MSI-high phenotype.

### SCNAs between tumors in subgroups 1, 2, and 3 categorized based on CNAs in IMNs with the MSI-high phenotype

**Fig. 4:**
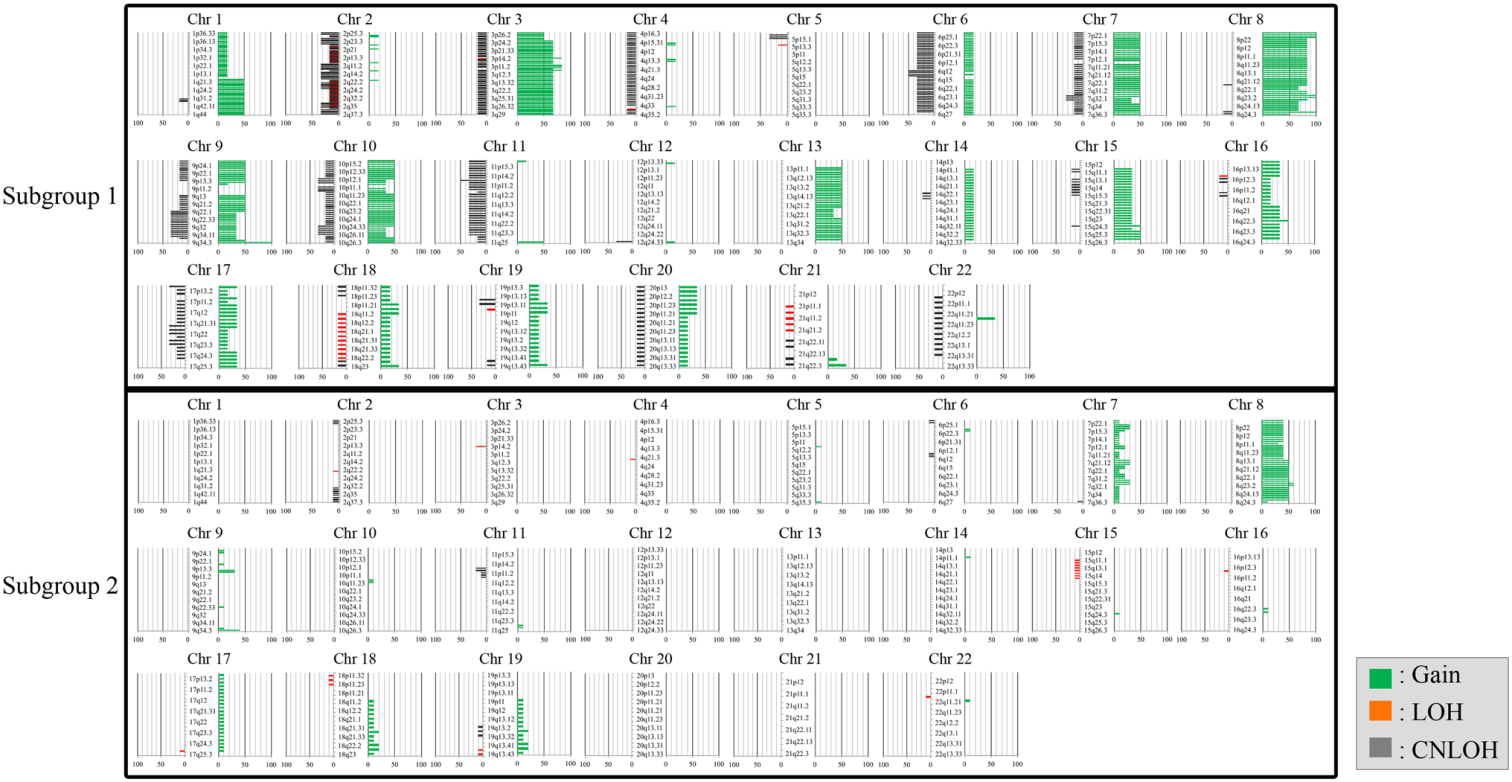
Ideogram of copy number alterations in two subgroups categorized based on somatic copy number alteration patterns in gastric intramucosal neoplasia with the MSI-high phenotype. Chromosomes are ordered from 1 to 22. The colored horizontal lines represent the frequencies of gains, LOHs, and CNLOHs. Lines on the left indicate losses (red, copy-neutral LOH; gray, LOH), and those on the right (green) indicate gains. **a** Ideogram of copy number alterations in subgroup 1. **b** Ideogram of copy number alterations in subgroup 2

The SCNAs of all chromosomes according to each subgroup are shown in Fig. 4a–c. The mean total number of chromosomal aberrations per patient was 321, with an average of 212 gains (range 103–273), 0 LOHs (range 0–49), and 101 copy-neutral LOHs (range 0–328) in subgroup 1. In addition, the mean total number of chromosomal aberrations per patient was 37, with an average of 32 gains (range 0–82), 2 LOHs (range 0–10), and 3 copy-neutral LOHs (range 0–10) in subgroup 2. There was a significant difference in the total number of SCNAs between subgroups 1 and 2 (*p* < 0.01; *p* < 0.001). Moreover, significant differences in the average number of copy number gains were found between subgroups 1 and 2 (*p* < 0.01; *p* < 0.01). LOH and copy-neutral LOH were not different in the two subgroups.

Regions of SCNAs detected in more than 30% of cases were selected. In subgroup 1, regions of gains were located at 1q, 3p, 3q, 7p, 7q, 8p, 8q, 9p, 9q21.11-q34.3, 10p, 10q, 13q, 15q, 16p12.3-p13.3, 16q21-q24.3, 17p11.2, 17p13.2-p13.3, 17q11.2-q21.33, 18q11.1-q12.1, and 20p, whereas those in subgroup 2 were at 7p21.1-p21.3, 7q21.11-21.13, 7q31.31-q31.33, 8p, 8q, and 9p12-p13.1 (in decreasing order of frequency). In addition, copy-neutral LOHs were found at 2p25.2-p25.3, 2q11.1-q12.3, 2q14.1-q21.2, 5p15.1-p15.31, 6p11.1-p25.2, 6q, 7q32.1-q32.3, 9q22.31-q34.2, 10p11.1-p11.22, 10q11.21-q21.1, 10q25.2-q26.13, 11p, 11q13.1-q24.3, 17p13.1, 17q22-q23.2, 17q24.1, and 19p11-p12 in subgroup 1 and at 7p21.1-p21.3, 7q21.11-21.13, 7q31.31-q31.33, 8p, 8q, and 9p12-p13.1 in subgroup 2; no regions of gains were found in subgroup 2.

### Differences in SCNAs between tumors in subgroups 1 and 2 categorized based on CNAs in IMN with the MSI phenotype

Finally, we examined differences in SCNAs between the three subgroups. Regions of gains detected in more than 50% of cases were selected for comparison of each group.

Significant differences in the frequencies of copy number gains between subgroups 1 and 2 were detected at 3p11.1-p12.1, 3p14.2, 3q11.2, 3p12.2-p14.1, 3p14.3-p24.3, 3q11.1, and 3q12.1-q29. No significant differences in losses (LOH and copy-neutral LOH) were observed between subgroups 1 and 2. In addition, LOH and CN LOH on 17p13.1 involving *TP53* were rare events (1 case in subgroup 1; no case in subgroup 2).

## Discussion

SCNAs have important roles in activating cancer-related genes, and an understanding of the biological and phenotypic effects of SCNAs may lead to substantial advances in cancer diagnostics and therapeutics [3]. Examination of SCNAs in tumor cells may provide information for predicting tumor aggressiveness [3] because SCNAs are closely associated with driver events that are acquired during cancer evolution [3, 21]. Determining how SCNAs promote the early phase of cancer is an important goal in human neoplasia. Thus, determination of SCNA patterns in IMNs may provide useful insight into early gastric carcinogenesis [21]. Moreover, microsatellite status (MSS or MSI) has been shown to be closely associated with molecular alterations in human neoplasia [9]. Thus, researchers are interested in examining the associations of SCNAs with microsatellite status in IMN. To the best of our knowledge, this study is the largest analysis to date of high-resolution copy number profiles of IMN specimens according to MSS and MSI.

In the present study, IMN with the MSS phenotype was stratified into three subgroups using a hierarchical clustering analysis performed based on SCNA patterns. Although tumors in subgroup 1 (6/84, 7.1%) were characterized by multiple SCNAs, tumors in subgroup 2 (12/84, 14.4%) were closely associated with a few SCNAs. Tumors in subgroup 1 were distinguished from those in subgroup 2 due to the frequent specific SCNAs (see Table 2). In contrast, tumors showing a few SCNAs were aggregated into subgroup 3, accounting for most of the IMNs examined in this study (78.6%). In addition, these findings supported that differences in the number of SCNAs may be regarded as the chromosomal destruction index. Moreover, although there are 3 genetic pathways according to SCNA patterns in early gastric carcinogenesis, the IMNs we examined could be largely classified into two categories, including high-frequency CNAs (subgroups 1 and 2) and low-frequency CNAs (subgroup 3). A previous study showed that copy number alterations arise as a result of preferential selection that favors cancer development [3, 22]. Our results suggested that this selection had already occurred by the early phase of GC.

Many LOHs at many chromosomal alleles, including 4q, 5q, 8p, and 9p, have been reported in previous studies [8, 19, 21. 22]. In the present study, however, the frequency of LOHs was low compared with those of previous studies, including those reported in The Cancer Genome Atlas, which is used as a common molecular Ref. [8]. Unfortunately, it is still unclear why there are differences in the frequencies of CNAs, including gains, LOH, and copy-neutral LOH, between the present and previous results. The first potential reason is that the platform used in the present study is different from that of previous studies [19]. Second, interstitial cells in the examined samples may influence the molecular analysis [23]. Indeed, LOH (loss of genetic material) is known to be affected by the dilution effect in interstitial cells contained in the sample rather than genetic gain [23]. Accordingly, CNA gains may be more easily detected than LOH. In the present study, however, we carefully confirmed that the samples we examined contain more than 50% tumor cells. In addition, a previous study showed that copy number gains are more common than losses across the entire genome in tumor tissues compared with paired normal tissues [24]. This study may support our results. Moreover, in the CNA gains we identified in the present study, 8q, which contains *c*-*myc*, may be a common and important chromosomal locus showing frequent gains in GCs. This finding may contribute to targeting the amplified “driver genes” in early gastric neoplasia. Finally, we believe that the high quality of the samples we examined was preserved.

Genetic mutations in tumors characterize the genetic features of the tumor cells [10]. Here, we did not find differences in the frequencies of genetic mutation (*KRAS*, *BRAF*, *PIK3CA* and *TP53*) between the three subgroups. However, previous studies have shown that *TP53* mutations are closely associated with early gastric carcinogenesis [25–28]. In a recent study, Fassan et al. showed the molecular similarity between high-grade intraepithelial neoplasia and early GC using a high-throughput mutation profiling method [29]. In addition, they suggested a relevant role for *TP53* mutations in early cancers. However, *TP53* mutations were rarely found in the IMNs examined in this study. The current findings suggested that chromosomal accumulation in intramucosal tumor cells played an important role in the development of intramucosal tumors rather than specific mutations, including *TP53* mutations.

The histological diagnosis of intramucosal neoplasia differs substantially between Western and Japanese pathologists [30–32]. This difference results most commonly from histological criteria of stromal invasion [6, 29–31]. The WHO histological classification of gastric neoplasia published at 2011 proposed new histological criteria for stromal invasion occurring in the mucosa propria [6, 31]. In the present study, LGD was more frequent in subgroup 3 than in subgroups 1 and 2, whereas IMC was significantly more frequent in tumors in subgroups 2 and 3 than in tumors in subgroup 1. However, no differences in the frequencies of HGD were found among the three subgroups. The associations of histological findings of IMNs with SCNAs have not been reported to date. Thus, LGD was characterized by a low frequency of SCNAs exhibiting an indolent nature, whereas IMC was closely associated with a high frequency of SCNAs showing a highly aggressive nature. Moreover, HGD was commonly observed in tumors in all subgroups. These findings validated the WHO classification of intramucosal neoplasia in terms of molecular alterations.

GC with the MSI-high phenotype is a distinct clinicopathological entity in gastric carcinogenesis [4, 5]. However, it is unclear whether the pathological and molecular concepts that have been identified in CRC with the MSI-high phenotype can be applied to GC with the MSI-high phenotype, particularly for IMNs with the MSI-high phenotype [4, 5]. Recent studies have shown that IMN with the MSI-high phenotype has multiple chromosomal alterations that are different from those of CRC with the MSI-high phenotype [4, 5]. In the present study, the molecular profile of IMN with the MSI-high phenotype was sub-classified into two subgroups based on SCNA patterns. Tumors in subgroup 1 were characterized by multiple SCNAs, whereas tumors in subgroup 2 were closely associated with a low frequency of SCNAs. In addition, these findings were supported by the observation of significant differences in the frequencies of SCNAs between subgroups 1 and 2. Additionally, significant differences in the frequencies of specific SCNAs (3p11.1-p12.1, 3p14.2, 3q11.2, 3p12.2-p14.1, 3p14.3-p24.3, 3q11.1, and 3q12.1-q29) were found between tumors in subgroups 1 and 2. However, to the best of our knowledge, in previous studies, no appropriate candidate genes located at these chromosomal loci showing amplification were identified in GCs. These findings suggested that there were two different subtypes in IMN with the MSI-high phenotype in terms of molecular profiles. In addition, our results suggested that there were different two subgroups showing distinct tumor characteristics, i.e., aggressive and indolent tumors. Interestingly, these findings demonstrated that there were distinct two molecular profiles in intramucosal neoplasia with the MSI phenotype.

In the present study, there were some limitations that may hinder application of the findings to clinical practice. Our results did not identify the clinicopathological differences between the subgroups. If the histological characteristics of IMNs based on each subgroup were identified, this information may be useful for routine histological diagnosis by pathologists. In addition, such studies will provide insights into determining the appropriate therapeutic plan and cutoff values of SCNAs quantified in IMNs. To the best of our knowledge, this is the first report analyzing SCNAs based on MSI and MSS in IMNs. Unfortunately, we could not validate the SCNA pattern in gastric IMNs using a second cohort. Further studies are needed to obtain this information.

In conclusion, we suggest that there are three subgroups based on SCNA patterns in IMN with the MSS phenotype. Tumors in subgroups 1 and 2 were characterized by multiple SCNAs, whereas those in subgroup 3 were characterized by a low frequency of SCNAs. Tumors in subgroup 1 could be distinguished from those in subgroup 2 in terms of specific SCNAs. These findings supported the concept that GC is a heterogeneous disease, even in the early phase (IMN) of gastric carcinogenesis. In contrast, our data showed that IMN with the MSI-high phenotype could be categorized into two subgroups based on SCNAs. This is the first study showing that a high frequency of SCNAs exists in IMN with the MSI phenotype. This concept will assist with histological diagnosis, endoscopic treatment, and therapeutic planning by providing novel insight into the mechanisms of early gastric carcinogenesis.

## Electronic supplementary material

Below is the link to the electronic supplementary material.

Supplementary material 1 (DOCX 16 kb)

Supplementary material 2 (DOCX 16 kb)

Supplementary material 3 (DOCX 16 kb)
