# Supplementary material for: Molecular profiling and comprehensive genome-wide analysis of somatic copy number alterations in gastric intramucosal neoplasias based on microsatellite status
Source: Gastric Cancer. 2018 Feb 21;21(5):765–75. doi: 10.1007/s10120-018-0810-5 (PMC6097076; doi:10.1007/s10120-018-0810-5)
Supplement: Supplementary file 1 — Supplementary material 1 (DOCX 16 kb) [file 10120_2018_810_MOESM1_ESM.docx]

Supplementary Table 1: Frequent SCNA regions in each subgroup in IMNs with the MSS phenotype

| Chromosomal regions | Subgroup 1 n = 6 (%) |  | Chromosomal regions | Subgroup 2 n = 12 (%) |
| --- | --- | --- | --- | --- |
| Gain |  |  | Gain |  |
| 8p, 8q、13q | 5-6 (83.3-100) |  | 5p, 5q | 7-12 (58.3-100) |
| 3p, 3q | 4-6 (66.7-100) |  | 17p, 17q | 4-11 (33.3-91.7) |
| 10p, 10q, 18p, 18q | 3-6 (50.0-100) |  | 20p, 20q | 7-10 (58.3-83.3) |
| 2p, 2q | 2-6 (33.3-100) |  | 9p | 4-10 (33.3-83.3) |
| 7p11.2-p22.3, 7q, 9q21.11-q34.3, 14q | 4-5 (66.7-83.3) |  | 18p, 18q | 4-9 (33.3-75.0) |
| 20p, 20q | 4-5 (66.7-83.3) |  | 10q | 6-7 (50.0-58.3) |
| 11p, 11q | 3-5 (50.0-83.3) |  | 1p, 6p, 6q | 4-7 (33.3-58.3) |
| 1q21.1-q44, 1p11.2-p13.3, 1p36.11-p36.23 | 2-5 (33.3-83.3) |  | 21q11.2-q22.13 | 5-6 (41.7-50.0) |
| 5p, 5q, 9p12-p24.3 | 2-5 (33.3-83.3) |  | 13q12.12-q13.3, 13q14.3-q34 | 4-6 (33.3-50.0) |
| 4p, 4q, 17p, 17q | 3-4 (50.0-66.7) |  | 15q11.2-q26.3, 19p12-p13.3 | 4-6 (33.3-50.0) |
| 6p, 6q, 12p, 12q, 16p, 16q | 2-4 (33.3-66.7) |  | 2p16.1-p16.3, 2q14.1-q35 | 4-5 (33.3-41.7) |
| 15q11.1-21.3, 15q22.31-q26.3, 19p | 2-3 (33.3-50.0) |  | 4q31.22-q32.2, 4q34.1-q35.2 | 4-5 (33.3-41.7) |
| CNLOH |  |  | 7p11.2-p22.3, 7q11.1-q21.11 | 4-5 (33.3-41.7) |
| 21q13.1 | 3 (50.0) |  | 7q21.13-q33, 7q35-q36.3, 14q | 4-5 (33.3-41.7) |
| 9p21.1-p21.3, 9p23-p24.3 | 2 (33.3) |  | 8p, 8q21.11, 8q21.3, 8q22.3-q24.11 | 4-5 (33.3-41.7) |
| 12q23.1-q24.31 | 2 (33.3) |  | 1q21.1-q21.3, 1q31.1-q32.1 | 4 (33.3) |
| 15q12-q13.1, 15q13.3-q24.1, 15q25.1-q26.3 | 2 (33.3) |  | CNLOH |  |
| 17p11.2-p13.3 | 2 (33.3) |  | None |  |
| 21q11.2-q21.3, 22q11.1-13.31 | 2 (33.3) |  | LOH |  |
| LOH |  |  | None |  |
| None |  |  |  |  |
|  |  |  | Chromosomal regions | Subgroup 3 n = 66 (%) |
|  |  |  | Gain |  |
|  |  |  | 9p13.1 | 21 (31.8) |
|  |  |  | 8q23.3 | 20 (30.3) |
|  |  |  | CNLOH |  |
|  |  |  | None |  |
|  |  |  | LOH |  |
|  |  |  | None |  |
