# Supplementary material for: Molecular profiling and comprehensive genome-wide analysis of somatic copy number alterations in gastric intramucosal neoplasias based on microsatellite status
Source: Gastric Cancer. 2018 Feb 21;21(5):765–75. doi: 10.1007/s10120-018-0810-5 (PMC6097076; doi:10.1007/s10120-018-0810-5)
Supplement: Supplementary file 2 — Supplementary material 2 (DOCX 16 kb) [file 10120_2018_810_MOESM2_ESM.docx]

Supplementary Table 2: Significant differences in the frequencies of SCNAs between subgroups 1 and 3 in IMNs with the MSS phenotype

|  | Subgroup 1 n = 6 (%) | Subgroup 3 n = 66 (%) | *p*-value |
| --- | --- | --- | --- |
| Gain |  |  |  |
| 18q | 5-6 (83.3-100) | 3-9 (4.5-13.6) | < 0.001 |
| 3p, 3q | 4-6 (66.7-100) | 2-8 (3.0-12.1) | < 0.001 |
| 10p, 10q11.21-q26.3 | 3-6 (50.0-100) | 0-4 (0-6.1) | < 0.001 |
| 2p12, 2p22.2-p25.3, 2q23.1-23.2, 2q36.1-q36.3 | 4-6 (66.7-100) | 2-7 (3.0-10.6) | < 0.001 |
| 1p11.2-p13.2, 1q32.1, 1q42.2-q44 | 3-5 (50.0-83.3) | 0-4 (0-6.1) | < 0.001 |
| 11p11.12-p14.2, 11p15.3-p15.5, 11q12.2-q25 | 3-5 (50.0-83.3) | 1-7 (1.5-10.6) | < 0.001 |
| 14q | 4-5 (66.7-83.3) | 1-2 (1.5-3.0) | < 0.001 |
| 9p22.3, 9q21.11-q34.3 | 4-5 (66.7-83.3) | 3-7 (4.5-10.6) | < 0.001 |
| 4p11-p15.33, 4p16.2, 4q21.21-21.22, 4q22.1-q24, 4q31.1-q31.21 | 3-4 (50.0-66.7) | 1-3 (1.5-4.5) | < 0.001 |
| 19p11 | 3 (50.0) | 0 | < 0.001 |
| 8p11.1-p21.3, 8p23.1-p23.3, 8q22.2, 8q24.11-q24.23 | 5-6 (83.3-100) | 8-11 (12.1-16.7) | < 0.001 |
| 13q11-q13.1, 13q14.11-q34 | 5-6 (83.3-100) | 7-14 (10.6-21.2) | < 0.001 |
| 5p11-p13.1, 5p14.1 | 5 (83.3) | 5-8 (7.6-12.1) | < 0.001 |
| 20p, 20q13.13-13.33 | 5 (83.3) | 6-8 (9.1-12.1) | < 0.001 |
| 6p11.1-p11.2, 6p12.2, 6q13, 6q16.3, 6q22.1-22.2 | 3-4 (50.0-66.7) | 1-3 (1.5-4.5) | < 0.001 |
| 12p12.2-p12.3, 12p13.2-p13.33 | 3-4 (50.0-66.7) | 3-4 (4.5-6.1) | < 0.001 |
| 17q11.1-q11.2, 17q21.31-q21.32, 17q22-q23.2 | 4 (66.7) | 3-4 (4.5-6.1) | < 0.001 |
| 16p11.1, 16p12.1-p13.11, 16p13.13, 16p13.2-13.3 | 3-4 (50.0-66.7) | 1-4 (1.5-6.1) | < 0.001 |
| 7p12.2-p13, 7q11.1-q11.23, 7q22.1-q31.2, 7q32.1-q34, 7q36.1-q36.3 | 5 (83.3) | 7-9 (10.6-13.6) | < 0.001 |
| 2p16.1-p16.3, 2q33.3-q35, 2q37.1-37.2 | 4 (66.7) | 5-6 (7.6-9.1) | < 0.001 |
| 11p14.3-p15.2, 11q11-q12.1 | 3-4 (50.0-66.7) | 2-6 (3.0-9.1) | < 0.01 |
| 12p11.1-p12.1, 12p13.1, 12q11, 12q14.1-21.33 | 3-4 (50.0-66.7) | 3-6 (4.5-9.1) | < 0.01 |
| 1q21.1-q23.1, 1q23.3-24.1, 1q25.3, 1q32.2-q42.13 | 3 (50.0) | 2-3 (3.0-4.5) | < 0.01 |
| 4p16.1, 4p16.3, 4q13.2-21.1, 4q21.23-q21.3, 4q25-q28.3, 4q31.22-q31.3 | 3 (50.0) | 2-4 (3.0-6.1) | < 0.01 |
| 6p12.1, 6p12.3-p21.1, 6p22.1-p22.2, 6q15-q16.1, 6q21, 6q23.1-q23.3 | 3 (50.0) | 2 (3.0) | < 0.01 |
| 7q21.11-q21.13, 7q21.2-21.3, 7q35, 8q11.1-13.3 | 5 (83.3) | 10 (15.2) | < 0.01 |
| 8q21.11-q21.3, 7p11.2-p12.1, 7q31.31-q31.32 | 5 (83.3) | 11 (16.7) | < 0.01 |
|  |  |  |  |
| CNLOH |  |  |  |
| 22q13.1 | 3 (83.3) | 1 (1.5) | < 0.01 |
| 9p21.1-p21.3, 9p23-p24.3 | 2 (33.3) | 0 | < 0.01 |
| 12q23.1-q24.31, 17p12-p13.3, 21q11.2-q21.3 | 2 (33.3) | 0 | < 0.01 |
| 15q13.3-q24.1, 15q25.1-q26.3, 22q11.21-q11.22, 22q12.2-q13.31 | 2 (33.3) | 1 (1.5) | < 0.05 |
|  |  |  |  |
| LOH |  |  |  |
| None |  |  |  |
