# Supplementary material for: Molecular profiling and comprehensive genome-wide analysis of somatic copy number alterations in gastric intramucosal neoplasias based on microsatellite status
Source: Gastric Cancer. 2018 Feb 21;21(5):765–75. doi: 10.1007/s10120-018-0810-5 (PMC6097076; doi:10.1007/s10120-018-0810-5)
Supplement: Supplementary file 3 — Supplementary material 3 (DOCX 16 kb) [file 10120_2018_810_MOESM3_ESM.docx]

Supplementary Table 3: Significant differences in the frequencies of SCNAs between subgroups 2 and 3 in IMNs with the MSS phenotype

| Gain | Subgroup 2 n = 12 (%) | Subgroup 3 n = 66 (%) | *p*-value |
| --- | --- | --- | --- |
| 5p, 5q11-q21.3, 5q23.3-q35.3 | 9-12 (75.0-100) | 5-14 (7.6-21.2) | < 0.001 |
| 17p, 17q11.2, 17q25.3 | 6-11 (50.0-91.7) | 4-10 (6.1-15.2) | < 0.001 |
| 9p13.2-p21.3, 9p23-p24.3 | 6-9 (50.0-75.0) | 4-9 (6.1-13.6) | < 0.001 |
| 10p13, 10q, 14q23.3-q24.1, 14q32.33 | 5-7 (41.7-58.3) | 0-4 (0-6.1) | < 0.001 |
| 18p11.21-p11.32 | 9 (75.0) | 5-6 (7.6-9.1) | < 0.001 |
| 20p12.1-p13, 20q11.21-q11.22, 20q12-q13.33 | 7-10 (58.3-83.3) | 6-8 (9.1-12.1) | < 0.001 |
| 1p31.2-p32.2, 1p33, 1p36.12, 1p36.21-p36.33 | 4-7 (33.3-58.3) | 0-4 (0-6.1) | < 0.001 |
| 6q11.1-q13, 6q16.1-q22.31, 6q22.33-q24.3, 6q26-q27 | 5-7 (41.7-58.3) | 1-4 (1.5-6.1) | < 0.001 |
| 15q24.1-q24.2, 15q25.2-q25.3, 21q21.1-q21.2 | 6 (50.0) | 4 (6.1) | < 0.001 |
| 9p22.1-p22.3, 20p11.21-p11.23, 20q11.22-q11.23 | 7 (58.3) | 7 (10.6) | < 0.01 |
| 1p22.1-p31.1, 1p32.3, 1p34.1-p34.3, 4p12-p13 | 4 (33.3) | 1 (1.5) | < 0.01 |
| 10p15.1-p15.3, 14q11.2-q32.31 | 4 (33.3) | 1 (1.5) | < 0.01 |
| 6p21.2, 15q24.3-q26.1, 18q22.1, 19q12 | 6 (50.0) | 5 (7.6) | < 0.01 |
| 1p35.2-p36.11, 6q14.1-q14.3, 6p11.1-p11.2 | 5 (41.7) | 3 (4.5) | < 0.01 |
| 17q22-q25.2, 18q12.2-q21.33, 21q22.12-q22.13 | 5 (41.7) | 3 (4.5) | < 0.01 |
| 10p11.21-p11.23 | 3 (25.0) | 0 | < 0.01 |
| 2q33.1-q33.2, 4q32.1, 6q15, 6p21.32-p21.33, 15q26.2-q26.3 | 5 (41.7) | 4 (6.1) | < 0.01 |
| 18q12.3, 18q21.2, 21q21.3-q21.11 | 5 (41.7) | 4 (6.1) | < 0.01 |
| 4q34.1-q34.2, 6q25.1-q25.2, 14q23.1-q23.2, 19q13.11-q13.12 | 4 (33.3) | 2 (3.0) | < 0.01 |
| 2q33.3-q35, 6p21.31, 6p22.1, 6p25.2, 17q24.3 | 5 (41.7) | 5 (7.6) | < 0.05 |
| 14p14-p15.32, 10p12.31-p12.33 | 3 (25.0) | 1 (1.5) | < 0.05 |
| 12p11.1-p11.21, 15q22.1-q22.32, 17q21.31-q21.33, 19p13.11-p13.13 | 4 (33.3) | 3 (4.5) | < 0.05 |
| 2q31.3-q32.2, 6p22.2, 6p25.3 | 5 (41.7) | 6 (9.1) | < 0.05 |
| CNLOH |  |  |  |
| 3p25.1-p26.1, 3q26.32-q28 | 3 (25.0) | 1 (1.5) | < 0.05 |
| 15q11.2, 15q13.2-q15.3, 15q22.2-q22.31 | 3 (25.0) | 1 (1.5) | < 0.05 |
| 17p11.2 | 3 (25.0) | 1 (1.5) | < 0.05 |
| LOH |  |  |  |
| None |  |  |  |
